# Reasons for Missing Antiretroviral Therapy: Results from a Multi-Country Study in Tanzania, Uganda, and Zambia

**DOI:** 10.1371/journal.pone.0147309

**Published:** 2016-01-20

**Authors:** Olivier Koole, Julie A Denison, Joris Menten, Sharon Tsui, Fred Wabwire-Mangen, Gideon Kwesigabo, Modest Mulenga, Andrew Auld, Simon Agolory, Ya Diul Mukadi, Eric van Praag, Kwasi Torpey, Seymour Williams, Jonathan Kaplan, Aaron Zee, David R Bangsberg, Robert Colebunders

**Affiliations:** 1 London School of Hygiene and Tropical Medicine, Department of Clinical Research, London, United Kingdom; 2 Institute of Tropical Medicine, Clinical Sciences Department, Antwerp, Belgium; 3 FHI 360, Social and Behavioral Health Sciences, Durham, North Carolina, United States of America; 4 Johns Hopkins Bloomberg School of Public Health, Department of International Health, Baltimore, Maryland, United States of America; 5 Infectious Diseases Institute, Makerere University College of Health Sciences, Kampala, Uganda; 6 Muhimbili University of Health and Allied Sciences, Dar es Salaam, United Republic of Tanzania; 7 Tropical Diseases Research Centre, Ndola, Zambia; 8 Division of Global HIV/AIDS, Centers for Disease Control and Prevention, Atlanta, Georgia, United States of America; 9 Massachusetts General Hospital, Boston, Massachusetts, United States of America; 10 Harvard Medical School, Boston, Massachusetts, United States of America; 11 Epidemiology and Social Medicine, University of Antwerp, Antwerp, Belgium; Azienda ospedaliero-universitaria di Perugia, ITALY

## Abstract

**Objectives:**

To identify the reasons patients miss taking their antiretroviral therapy (ART) and the proportion who miss their ART because of symptoms; and to explore the association between symptoms and incomplete adherence.

**Methods:**

Secondary analysis of data collected during a cross-sectional study that examined ART adherence among adults from 18 purposefully selected sites in Tanzania, Uganda, and Zambia. We interviewed 250 systematically selected patients per facility (≥18 years) on reasons for missing ART and symptoms they had experienced (using the HIV Symptom Index). We abstracted clinical data from the patients’ medical, pharmacy, and laboratory records. Incomplete adherence was defined as having missed ART for at least 48 consecutive hours during the past 3 months.

**Results:**

Twenty-nine percent of participants reported at least one reason for having ever missed ART (1278/4425). The most frequent reason was simply forgetting (681/1278 or 53%), followed by ART-related hunger or not having enough food (30%), and symptoms (12%). The median number of symptoms reported by participants was 4 (IQR: 2–7). Every additional symptom increased the odds of incomplete adherence by 12% (OR: 1.1, 95% CI: 1.1–1.2). Female participants and participants initiated on a regimen containing stavudine were more likely to report greater numbers of symptoms.

**Conclusions:**

Symptoms were a common reason for missing ART, together with simply forgetting and food insecurity. A combination of ART regimens with fewer side effects, use of mobile phone text message reminders, and integration of food supplementation and livelihood programmes into HIV programmes, have the potential to decrease missed ART and hence to improve adherence and the outcomes of ART programmes.

## Introduction

At the end of 2013 two-thirds of the estimated 35 million people globally living with HIV lived in sub-Saharan Africa.[1] The number of people receiving antiretroviral treatment (ART) reached about 13 million. Sub-Saharan Africa achieved the greatest increase in ART coverage by reaching 9 million people, corresponding to about 37% coverage among people living with HIV in that region.[1,2] The goal of ART is to achieve and sustain viral suppression to achieve the full clinical and prevention benefits of HIV treatment.[3,4] A systematic review of studies from low-and middle income countries reported a pooled estimate of viral suppression (<1000 copies/ml) of 78% (95% confidence interval (95% CI):68%-86%) at 12 months after ART initiation.[5]

Achieving viral suppression requires consistent adherence to ART.[6] Factors identified in the literature as affecting ART adherence include patient characteristics (socio-demographic and psychosocial factors), patient/provider aspects (patient-provider interactions, trust, and confidentiality), health-system related factors (waiting time at the clinic, transport), disease characteristics (HIV-related symptoms), and therapy-related factors (number of pills, medication side effects).[7,8]

In order to develop effective adherence interventions, it is important to identify the common reasons people report for not taking their ART. We used data collected during a cross-sectional study conducted in 2011 that examined adherence to ART among adults in three countries in sub-Saharan Africa: Tanzania, Uganda, and Zambia.[9] In the primary paper we examined individual and programmatic factors associated with incomplete adherence. We found that 3% of participants missed two or more consecutive days of their ART in the past three months, and that having greater versus less self-reported HIV-related symptoms (a dichotomized variable based on the country-specific median number of symptoms) was significantly associated with incomplete adherence. In this secondary analysis we focused on self-reported reasons for ever missing ART and investigated the role of symptoms in missing ART. We also explored the association between having experienced specific symptoms and incomplete adherence.

## Methods

### Design and study setting

From May to October 2011, a cross-sectional study was conducted among ART patients from 18 purposively selected study sites in Tanzania, Uganda, and Zambia. Site selection has been described in an earlier publication [10] and included clinics from different levels in the health system (ranging from rural health centres to referral hospitals), from different types of health facilities (public sector, non-governmental organizations (NGOs), or faith-based organizations), and with different ART provision experiences and adherence strategies.

### Inclusion criteria

Patients attending the study sites who were at least 18 years of age at ART initiation, who initiated ART at least six months prior to the interview, and who spoke one of the study languages were eligible for inclusion.

### Data collection and sampling

Based on clinic client flow, participants were selected using a systematic sampling approach with every fifth patient selected at the larger facilities and every third patient at the smaller clinics. After selection, potential participants were screened for eligibility by trained research interviewers, and, if they consented, interviewed until we obtained a sample of 250 eligible patients from each facility (4500 patients in total).

### Measures

The survey was designed in English and then translated into 10 languages (Swahili for Tanzania; Acholi, Luganda, Lumasaaba, Lunyankore for Uganda; and Bemba, Chewa, Lozi, Nyanja and Tonga for Zambia) and pretested during the training of the fieldworkers and piloting of the study-instruments.

The survey contained several measures on self-reported adherence, and questions on psychosocial factors including stigma (Internalized Stigma Scale [11]), depression (Hopkins Symptoms Checklist [12]), social support (Duke University Functional Social Support questionnaire [13]), and alcohol abuse (CAGE [14]).

The survey also included a list of 16 reasons for ever missing ART (based on the AACTG questionnaire) [15] and on symptoms experienced in the four weeks prior to the interview. Symptoms were collected using a modified HIV Symptom Index that has 20 items scored on a five point Likert scale.[16] During translation and pre-testing the team modified the responses from a five point to a four point Likert scale, with 0 representing the absence of that symptom and 3 indicating that the patient did have the symptom and it bothered them “a lot” resulting in an index that ranges from 0 to 60. The participant was also asked if they attributed the symptom to their ART.

Based on previous evidence of the importance of treatment interruptions as a predictor of viral load failure and resistance [17,18], and because missing ART for at least 48 consecutive hours was the strongest measure related to virological failure during our primary analysis, we constructed a missed at least 48 consecutive hours measure from two questions about missed tablets in the past 3 months to define incomplete adherence.[9]

Data regarding ART initiation (ART start dates and regimens, and pre-ART characteristics) were abstracted from the patient’s medical, pharmacy, and laboratory records using structured data abstraction forms.

### Data management and analysis

All data were double entered in a study database using EpiData Entry 3.1 (EpiData Association, Odense, Denmark) at the in-country research organizations, and then transferred to the central data office at Family Health International 360 (FHI 360) for further cleaning and consistency checks.

For data analysis, the proportions of reasons for ever missing one’s ART and experiences of symptoms in the past four weeks are reported. Because of the dependency on self-reported symptoms, we investigated the relationship between symptoms and incomplete adherence in the past three months as a purely explorative objective, and therefore limited this analysis to univariate analysis only. We also explored the association between specific antiretroviral drugs (nevirapine versus efavirenz, and stavudine versus zidovudine) and symptom burden (expressed as the HIV Symptom Index score) using multiple linear regression, with backwards stepwise elimination, including site as a fixed effect. Variables associated with symptom burden with a p-value <0.10 were considered for multivariable analysis, and variables with a p-value <0.05 in the multivariable analysis were considered significant.

### Ethics statement

The study was reviewed and approved by the institutional review board (IRB) of the U.S. Centers for Disease Control and Prevention (CDC) and the six partner and national ethical review committees. The Partners Healthcare IRB ceded review to FHI 360.

## Results

A total of 6825 patients were screened for eligibility at the participating sites. Of these 1848 patients were ineligible, and 482 did not provide informed consent. An additional 70 patients with no data on missed ART for at least 48 consecutive hours were excluded, leaving 4425 patients for the final analysis.

### Characteristics of study population

Characteristics at the time of the interview and at ART initiation, stratified by country, are presented in Table 1. Participants were predominantly female (68%) and had started ART between 2002 and 2011. At ART initiation the median age was 40 years (inter quartile range (IQR): 34–47 years) and the median CD4 cell count was 145 cells/μl (IQR: 75–217). At the time of the interview, the median time on ART was 4 years (IQR: 2–5 years) and about 45% of participants had changed their ART regimen since initiation. Twenty-three percent changed from an initial stavudine (d4T) containing regimen, 13% from an initial nevirapine (NVP) containing regimen and 7% from an initial efavirenz (EFV) containing regimen. One-third of patients (32%) were receiving AZT/3TC/NVP, 17% AZT/3TC/EFV, and 16% d4T/3TC/NVP or TDF/3TC-FTC/EFV at the time of data collection. About 3% of patients were receiving a second-line regimen that contained a protease inhibitor.

**Table 1:** Characteristics at interview and at ART initiation of study population in multi-country (Tanzania, Uganda, and Zambia) adherence study, 2011.

Characteristic	Tanzania (n = 1469)	Uganda (n = 1474)	Zambia (n = 1482)	Total number of patients (n = 4425)
**Gender: n (%)**				
**Male**	394 (26.8)	505 (34.3)	520 (35.1)	1419 (32.1)
**Female**	1075 (73.2)	969 (65.7)	962 (64.9)	3006 (67.9)
**At interview**				
**Age (years): median (IQR)**	41 (35–47)	39 (34–46)	40 (34–47)	40 (34–47)
**Years on ART: median (IQR)**	3.2 (2.0–4.6)	3.6 (2.2–5.4)	4.2 (2.5–5.7)	3.6 (2.2–5.3)
**CD4 (cells/μL): median (IQR)**	372 (243–548)	368 (245–524)	427 (291–588)	391 (255–560)
**Missing: n (%)**	701 (47.7)	803 (54.5)	700 (47.2)	2204 (49.8)
**ART regimen: n (%)**				
**d4T-3TC-NVP**	549 (37.4)	11 (0.7)	158 (10.7)	718 (16.2)
**AZT-3TC-EFV**	389 (26.5)	296 (20.1)	76 (5.1)	761 (17.2)
**AZT-3TC-NVP**	258 (17.6)	898 (60.9)	253 (17.1)	1409 (31.8)
**TDF-3TC/FTC-EFV**	114 (7.8)	83 (5.6)	493 (33.3)	690 (15.6)
**PI-containing**	31 (2.1)	39 (2.6)	66 (4.5)	136 (3.1)
**Other**	38 (2.6)	134 (9.1)	369 (24.9)	541 (12.2)
**Missing**	90 (6.1)	13 (0.9)	67 (4.5)	170 (3.8)
**Regimen containing: n (%)**				
**EFV**	517 (35.2)	382 (25.9)	641 (43.3)	1540 (34.8)
**NVP**	823 (56.0)	1036 (70.3)	708 (47.8)	2567 (58.0)
**d4T**	575 (39.1)	23 (1.6)	207 (14.0)	805 (18.2)
**AZT**	660 (44.9)	1217 (82.6)	347 (23.4)	2224 (50.3)
**TDF**	123 (8.4)	221 (15.0)	787 (53.1)	1131 (25.6)
**Regimen change since ART initiation: n (%)**				
**Yes**	663 (45.1)	667 (45.3)	670 (45.2)	2000 (45.2)
**No**	806 (54.9)	807 (54.7)	812 (54.8)	2425 (54.8)
**Change from NVP at initiation: n (%)**				
**Yes**	246 (16.7)	141 (9.6)	197 (13.3)	584 (13.2)
**No**	708 (48.2)	924 (62.7)	504 (34.0)	2136 (48.3)
**Not applicable**	515 (35.1)	409 (27.7)	781 (52.7)	1705 (38.5)
**Change from EFV at initiation: n (%)**				
**Yes**	165 (11.2)	59 (4.0)	64 (4.3)	288 (6.5)
**No**	342 (23.3)	259 (17.6)	495 (33.4)	1096 (24.8)
**Not applicable**	962 (65.5)	1156 (78.4)	923 (62.3)	3041 (68.7)
**Change from d4T at initiation: n (%)**				
**Yes**	345 (23.5)	400 (27.1)	256 (17.3)	1001 (22.6)
**No**	442 (30.1)	6 (0.4)	183 (12.3)	631 (14.3)
**Not applicable**	682 (46.4)	1068 (72.5)	1043 (70.4)	2793 (63.1)
**Change from AZT at initiation: n (%)**				
**Yes**	212 (14.4)	82 (5.6)	110 (7.4)	404 (9.1)
**No**	427 (29.1)	840 (57.0)	256 (17.3)	1523 (34.4)
**Not applicable**	830 (56.5)	552 (37.4)	1116 (75.3)	2498 (56.5)
**At ART initiation**				
**Year of ART initiation: n (%)**				
**2002–2004**	10 (0.7)	60 (4.1)	97 (6.5)	167 (3.8)
**2005**	123 (8.4)	180 (12.2)	192 (13.0)	495 (11.2)
**2006**	155 (10.6)	152 (10.3)	209 (14.1)	516 (11.7)
**2007**	198 (13.5)	224 (15.2)	254 (17.1)	676 (15.3)
**2008**	272 (18.5)	219 (14.9)	241 (16.3)	732 (16.5)
**2009**	315 (21.4)	280 (19.0)	235 (15.9)	830 (18.8)
**2010**	356 (24.2)	313 (21.2)	240 (16.2)	909 (20.5)
**2011**	36 (2.5)	43 (2.9)	11 (0.7)	90 (2.0)
**Missing**	4 (0.2)	3 (0.2)	3 (0.2)	10 (0.2)
**WHO clinical stage: n (%)**				
**Stage 1 and 2**	414 (28.2)	704 (47.8)	567 (38.3)	1685 (38.1)
**Stage 3**	659 (44.9)	512 (34.7)	671 (45.3)	1842 (41.6)
**Stage 4**	274 (18.6)	151 (10.2)	92 (6.2)	517 (11.7)
**Missing**	122 (8.3)	107 (7.3)	152 (10.2)	381 (8.6)
**CD4 (cells/μL): median (IQR)**	138 (68–218)	149 (83–211)	147 (75–228)	145 (75–217)
**Missing: n (%)**	281 (19.3)	296 (20.1)	312 (21.1)	889 (20.1)
**ART regimen: n (%)**				
**d4T-3TC-NVP**	736 (50.1)	394 (26.7)	396 (26.7)	1526 (34.5)
**AZT-3TC-EFV**	421 (28.7)	251 (17.0)	77 (5.2)	749 (16.9)
**AZT-3TC-NVP**	218 (14.8)	668 (45.3)	288 (19.5)	1174 (26.5)
**TDF-3TC/FTC-EFV**	36 (2.5)	54 (3.7)	403 (27.2)	493 (11.1)
**PI-containing**	5 (0.3)	16 (1.1)	12 (0.8)	33 (0.8)
**Other**	51 (3.5)	13 (0.9)	95 (6.4)	159 (3.6)
**Missing**	2 (0.1)	78 (5.3)	211 (14.2)	291 (6.6)
**Regimen containing: n (%)**				
**EFV**	507 (34.5)	318 (21.6)	559 (37.7)	1384 (31.3)
**NVP**	954 (64.9)	1065 (72.3)	701 (47.3)	2720 (61.5)
**d4T**	787 (53.6)	406 (27.5)	439 (29.6)	1632 (36.9)
**AZT**	639 (43.5)	922 (62.6)	366 (24.7)	1927 (43.6)
**TDF**	36 (2.5)	63 (4.3)	409 (27.6)	508 (11.5)

d4T: stavudine; 3TC: lamivudine; NVP: nevirapine; EFV: efavirenz; AZT: zidovudine; TDF: tenofovir; FTC: emtricitabine; PI: protease inhibitor.

### Incomplete adherence (defined as missed ART for at least 48 consecutive hours during the past 3 months)

About 3% (141/4425) of our study participants had missed taking their ART for at least 48 consecutive hours during the past 3 months.

### Reasons for ever missing one’s ART

**Fig 1:**
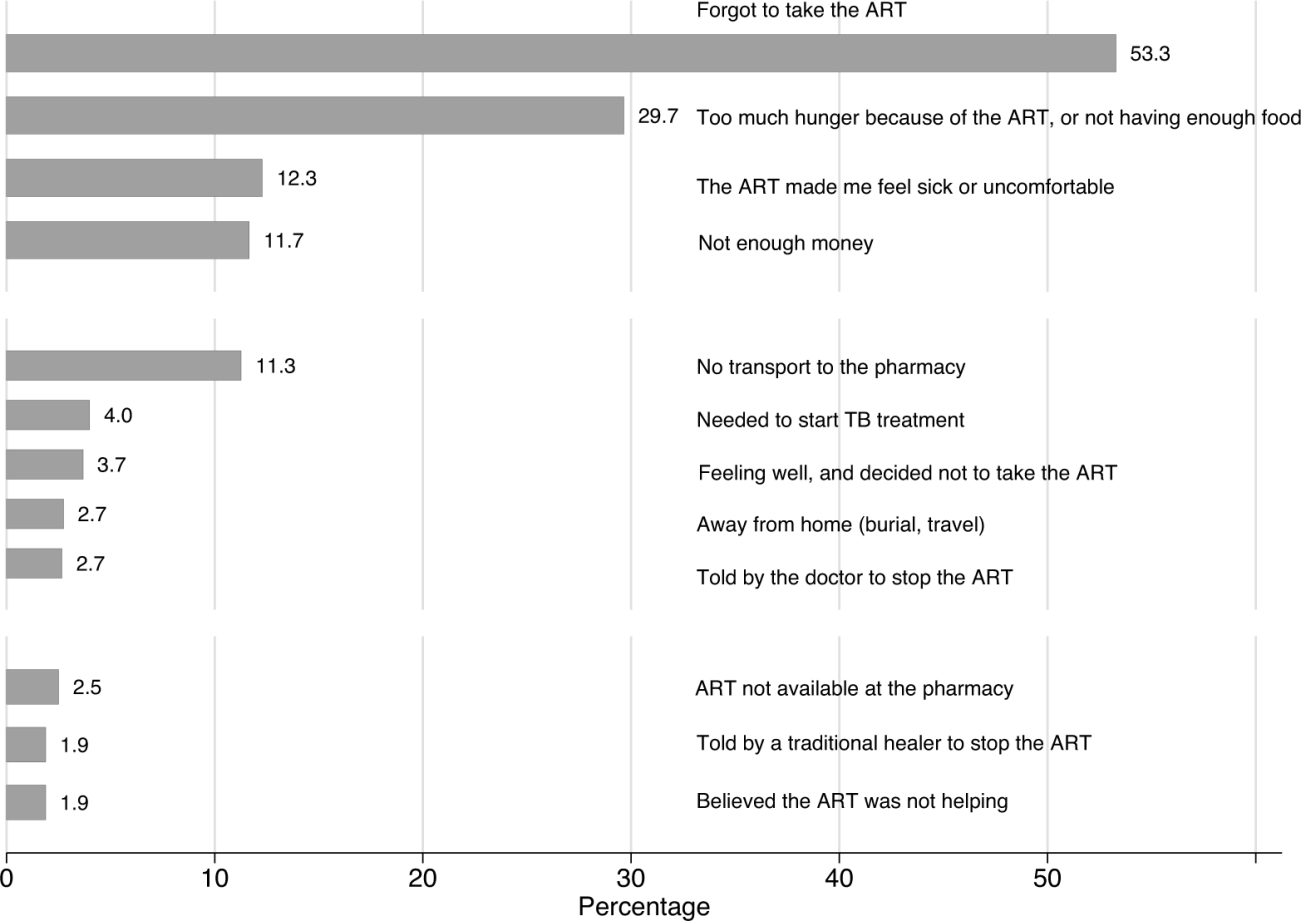
Reasons for patients who ever missed ART in multi-country (Tanzania, Uganda, and Zambia) adherence study (N = 1278), 2011.

Among all ART patients, 29% of participants reported at least one reason for having ever missed ART (1278/4425), with only 0.2% (9/4425) missing responses to these questions. About half of patients who reported ever missing ART (53% or 681/1278) reported they simply forgot, followed by having too much hunger because of ART or not having enough food (30%), and feeling sick or uncomfortable because of the ART (12%) (Fig 1). Other frequently cited reasons included no transport to the pharmacy (11%) and being away from home (3%).

A small number of patients (about 2% of those who ever missed ART or less than 1% of all patients in the study) have ever missed taking their ART because they were told to stop taking the drugs by a traditional healer. Of the 4,425 patients in this study, 257 (6%) ever consulted a traditional healer or herbalist because of HIV, the majority of whom were from Tanzania (76%).

### HIV Symptom Index Score (symptom burden)

Eighty-eight percent of participants reported experiencing at least one symptom during the past four weeks (Table 2), with responses missing for only a small proportion of participants (5/4425). The median number of symptoms reported was 4 (IQR: 2–7) with a median HIV Symptom Index score of 8 (IQR: 3–14). Women reported significantly more symptoms (median of 4 among women versus 3 among men) and a higher symptom burden (median HIV Symptom Index score of 8 among women versus 7 among men). More symptoms [5 (IQR: 3–8)] and a higher median HIV Symptom Index score [11 (IQR: 5–17)] were reported in Uganda (Kruskal Wallis p-value<0.001 for both comparisons).

**Table 2:** Reported symptoms in the past four weeks in multi-country (Tanzania, Uganda, and Zambia) adherence study, 2011.

	Tanzania (n = 1469)	Uganda (n = 1474)	Zambia (n = 1482)	Total (n = 4425)	Attributed to ARVs by patient (n, %)	Severe symptoms (“It bothers me a lot”) (n, %)
Reporting at least one symptom, n (%)	1193 (81.2)	1389 (94.2)	1325 (89.4)	3907 (88.3)		
Number of symptoms reported, median (IQR)	3 (1–6)	5 (3–8)	4 (2–6)	4 (2–7)		
HIV Symptom Index score, median (IQR)	6 (2–12)	11 (5–17)	7 (3–13)	8 (3–14)		
Symptom, n (%)						
Fatigue or loss of energy	360 (24.5)	754 (51.2)	514 (34.7)	1628 (36.8)	308 (18.9)	534 (32.8)
Pain, numbness, or tingling in the hands or feet	462 (31.4)	662 (44.9)	436 (29.4)	1560 (35.3)	512 (32.8)	583 (37.4)
Headache	394 (26.8)	610 (41.4)	515 (34.8)	1519 (34.3)	233 (15.3)	415 (27.3)
Felt sad, down or depressed	451 (30.7)	518 (35.1)	340 (22.9)	1309 (29.6)	140 (10.7)	467 (35.7)
Muscle aches or joint pains	382 (26.0)	524 (35.5)	284 (19.2)	1190 (26.9)	314 (26.4)	435 (36.6)
Fat deposits or weight gain	378 (25.7)	289 (19.6)	480 (32.4)	1147 (25.9)	326 (28.4)	145 (12.6)
Fevers, chills, or sweats	237 (16.1)	524 (35.5)	374 (25.2)	1135 (25.6)	178 (15.7)	333 (29.3)
Trouble remembering	220 (15.0)	470 (31.9)	419 (28.3)	1109 (25.1)	405 (36.5)	400 (36.1)
Cough or breathing difficulties	208 (14.2)	488 (33.1)	330 (22.3)	1026 (23.2)	128 (12.5)	326 (31.8)
Problems with having sex (such as loss of interest or a lack of satisfaction)	316 (21.5)	388 (26.3)	312 (21.1)	1016 (23.0)	317 (31.2)	423 (41.6)
Weight loss or wasting	278 (18.9)	411 (27.9)	321 (21.7)	1010 (22.8)	167 (16.5)	267 (26.4)
Dizzy or lightheaded	250 (17.0)	429 (29.1)	320 (21.6)	999 (22.6)	287 (28.7)	267 (26.7)
Loss of appetite or change in taste of food	267 (18.2)	341 (23.1)	307 (20.7)	915 (20.7)	252 (27.5)	318 (34.8)
Skin problems (rash, dryness, or itching)	257 (17.5)	381 (25.8)	205 (13.8)	843 (19.1)	255 (30.2)	369 (43.8)
Difficulty falling or staying asleep	259 (17.6)	327 (22.2)	251 (16.9)	837 (18.9)	204 (24.4)	306 (36.6)
Felt nervous or anxious	248 (16.9)	373 (25.3)	191 (12.9)	812 (18.4)	117 (14.4)	271 (33.4)
Bloating, stomach pain, or gas	249 (17.0)	353 (23.9)	194 (13.1)	796 (18.0)	219 (27.5)	265 (33.3)
Nausea or vomiting	132 (9.0)	203 (13.8)	189 (12.8)	524 (11.8)	176 (33.6)	147 (28.1)
Diarrhea or loose bowl movements	148 (10.1)	146 (9.9)	181 (12.2)	475 (10.7)	100 (21.1)	122 (25.7)
Hair loss or hair change	36 (2.5)	101 (6.9)	42 (2.8)	179 (4.0)	63 (35.2)	60 (33.5)

Female gender and being initiated on a d4T—or NVP—containing ART regimens were associated with having more symptoms during univariate analysis (Table 3). During multivariable analysis, females (coefficient: +1.6, 95%CI: +1.0, +2.2) and participants taking ART regimens containing d4T at initiation (coefficient: +0.9, 95% CI: +0.3, +1.4) remained significantly associated with a greater symptom burden.

**Table 3:** Regression coefficients of factors associated with symptom burden (expressed as HIV Symptom Index Score) in multi-country (Tanzania, Uganda, and Zambia) adherence study, 2011.

Risk factor	Single regression*		Multiple regression*	
	Coefficient (95% CI)	P-value	Coefficient (95% CI)	P-value
Male (versus female)	-1.6 (-2.2, -1.0)	<0.001	-1.6 (-2.2, -1.0)	<0.001
WHO clinical stage at ART initiation (versus WHO stage 1 and 2)		0.138		
Stage 3	+0.7 (+0.1, +1.4)			
Stage 4	+0.6 (-0.3, +1.6)			
Missing	+0.7 (-0.3, +1.8)			
CD4 cell count at ART initiation (versus ≥250 cells/μL)		0.495		
< 250 cells/μL	-0.1 (-1.4, +0.4)			
Missing	-0.5 (-1.6, +0.5)			
Regimen at ART initiation containing				
NVP (versus EFV)	+1.0 (+0.3, +1.6)	0.006		NS
d4T (versus AZT)	+0.9 (+0.3, +1.4)	0.003	+0.9 (+0.3, +1.4)	0.004
Current age (per 10 years)	-0.1 (-0.4, +0.2)	0.479		
Duration on ART (per year)	+0.1 (-0.1, +0.2)	0.400		
CD4 cell count (versus ≥250 cells/μL)		0.367		
< 250 cells/μL	+0.6 (-0.3, +1.5)			
Missing	-0.1 (-0.7, +0.6)			
Current regimen containing				
NVP (versus EFV)	+0.6 (-0.1, +1.3)	0.115		
d4T (versus AZT)	+0.7 (-0.2, +1.5)	0.109		

*Regression coefficients and P-values calculated using linear regression with site as a fixed effect.

Factors with negative coefficients are associated with a reduced symptom burden; those with a positive coefficient with an increased symptom burden.

d4T: stavudine; NVP: nevirapine; EFV: efavirenz; AZT: zidovudine; CI: confidence interval.

### Types of symptoms

Fatigue or loss of energy was the most cited symptom (37%), followed by pain, numbness, or tingling in the hands or feet (35%), and headache (34%). Feeling sad, down, or depressed was reported by 30% of participants (Table 2). Of these most frequently reported symptoms, only pain, numbness, or tingling in the hands or feet was attributed to ART by a substantial proportion of participants with this symptom (33%). Other symptoms that were not as common but more frequently attributed to ART by at least 30% of participants with that symptom included trouble remembering (37%), hair loss or changes in hair (35%), nausea or vomiting (34%), problems with having sex (31%), and skin problems (30%).

Men reported significantly more diarrhoea, skin problems, and problems with having sex while women reported significantly more fatigue or loss of energy; headache; loss of appetite or change in the taste of food; fever, chills, or sweats; feeling dizzy or lightheaded; troubles remembering; nausea or vomiting; feeling sad, down, or depressed; weight loss or wasting; hair loss or hair change; bloating, pain, or gas; and muscle aches or joint pains (data not shown).

### Association between symptoms and incomplete adherence

Every additional symptom increased the odds of incomplete adherence by 10% (odds ratio (OR): 1.10, 95% CI: 1.05–1.15) (data not shown). Patients who reported at least one symptom were more likely to have incomplete adherence (OR: 3.0, 95% CI: 1.2–7.4) (Table 4). About half of the symptoms from the HIV Symptom Index Score (9/20) were associated with incomplete adherence: fatigue or loss of energy (OR: 1.5, 95% CI: 1.0–2.1), fevers, chills, or sweats (OR: 1.7, 95% CI: 1.2–2.4), nausea or vomiting (OR: 1.7, 95% CI: 1.1–2.7), diarrhoea or loose bowl movements (OR: 2.2, 95% CI: 1.5–3.4), feeling sad, down, or depressed (OR: 1.5, 95% CI: 1.1–2.1), skin problems (OR: 2.0, 95% CI: 1.4–2.8), cough or breathing difficulties (OR: 1.8, 95% CI: 1.3–2.6), loss of appetite or change in the taste of food (OR: 2.1, 95% CI: 1.4–3.0), and abdominal pains (OR: 1.6, 95% CI: 1.1–2.3).

**Table 4:** Analysis of individual symptoms with incomplete adherence as outcome (defined as missed ART for at least 48 consecutive hours during the past 3 months) in multi-country (Tanzania, Uganda, and Zambia) adherence study, 2011.

	Total (n = 4425)	Incomplete adherence (n = 141)	Crude Odds Ratio (OR) (95% CI) *	P-value*
Reporting at least one symptom, n (%)	3907 (88.3)	136 (96.5)	2.95 (1.19–7.35)	0.007
Symptom, n (%)				
Fatigue or loss of energy	1628 (36.8)	67 (47.5)	1.45 (1.01–2.06)	0.042
Pain, numbness, or tingling in the hands or feet	1560 (35.3)	57 (40.4)	1.11 (0.78–1.58)	0.562
Headache	1519 (34.3)	61 (43.3)	1.29 (0.91–1.83)	0.162
Felt sad, down or depressed	1309 (29.6)	63 (44.7)	1.49 (1.05–2.12)	0.029
Muscle aches or joint pains	1190 (26.9)	49 (34.8)	1.25 (0.87–1.80)	0.240
Fat deposits or weight gain	1147 (25.9)	37 (26.2)	0.99 (0.66–1.47)	0.950
Fevers, chills, or sweats	1135 (25.6)	52 (36.9)	1.67 (1.16–2.41)	0.007
Trouble remembering	1109 (25.1)	37 (26.2)	1.02 (0.69–1.52)	0.906
Cough or breathing difficulties	1026 (23.2)	51 (36.2)	1.80 (1.25–2.61)	0.002
Problems with having sex (such as loss of interest or a lack of satisfaction)	1016 (23.0)	39 (27.7)	1.13 (0.76–1.66)	0.554
Weight loss or wasting	1010 (22.8)	39 (27.7)	1.20 (0.81–1.77)	0.362
Dizzy or lightheaded	999 (22.6)	45 (31.9)	1.42 (0.98–2.06)	0.071
Loss of appetite or change in taste of food	915 (20.7)	53 (37.6)	2.05 (1.43–2.95)	<0.001
Skin problems (rash, dryness, or itching)	843 (19.1)	48 (34.0)	1.95 (1.35–2.81)	<0.001
Difficulty falling or staying asleep	837 (18.9)	38 (27.0)	1.35 (0.91–1.99)	0.145
Felt nervous or anxious	812 (18.4)	38 (27.0)	1.27 (0.85–1.89)	0.256
Bloating, stomach pain, or gas	796 (18.0)	40 (28.4)	1.59 (1.08–2.34)	0.022
Nausea or vomiting	524 (11.8)	28 (19.9)	1.72 (1.11–2.67)	0.020
Diarrhoea or loose bowl movements	475 (10.7)	31 (22.0)	2.24 (1.47–3.43)	<0.001
Hair loss or hair change	179 (4.0)	6 (4.3)	1.01 (0.43–2.36)	0.975

*OR and P-value calculated using logistic regression with site as a fixed effect.

OR: odds ratio; CI: confidence interval.

Patients who reported feeling sick and uncomfortable because of ART were also more likely to have incomplete adherence (OR: 3.7, 95% CI: 2.1–6.6).

## Discussion

This is the first study to our knowledge to interview more than 4000 patients on their reasons for ever missing ART and their current experiences with HIV-related symptoms using a consistent data collection tool across 18 study sites in 3 countries in sub-Saharan Africa. This paper builds on the study’s primary analysis that found that 3% of participants missed two or more consecutive days of their ART in the past three months and that having greater numbers of self-reported symptoms, (defined as more than the country-specific median), was significantly related to this measure of incomplete adherence.[9] In this secondary analysis we focused on specific symptoms and their association with incomplete adherence, and on reasons for ever missing ART.

This analysis found that about one third of participants (29%) reported ever missing ART. Simply forgetting was cited as the most common reason for ever missing ART, a result which concurs with findings from other studies.[19–21] Advances in mHealth technologies are emerging as an option to address forgetting to take ART. For example, several studies have found that patients who received text messages have better levels of ART adherence and clinical indicators, such as lower viral loads and higher CD4 cell counts, compared to patients who did not receive text messages.[22–24] However, in their network meta-analysis examining direct and indirect evidence from randomized trials, Mills and colleagues found a large benefit for weekly but not for daily SMS messages [25], emphasizing the need for tailored mHealth interventions.[26] Such findings support the inclusion of mobile phone text messaging in the package of adherence intervention tools, as recommended by the World Health Organization in their latest guidelines, as well as the need for more research on how to optimize the use of text messaging.[27] However, the potential of unwanted disclosure if the message is intercepted by others, and the cost and sustainability of these mHealth interventions in the absence of external funding, remain important issues.[28]

Having too much hunger because of ART or not having enough food was experienced by about one-third of participants who reported ever missing ART. The mechanism of ART-related hunger is unclear, but the possibility of immunological phenomena may play a role.[29] Food insecurity has been described as an important barrier to adherence and subsequent mortality in impoverished populations.[30–35] The integration of food supplementation into HIV care programmes has been shown to improve adherence [36], and to have clinical and immunological benefits.[37] In order to address the underlying causes of food insecurity, organizations are looking for more sustainable long-term solutions to address this issue in the form of livelihood programmes.[38] The lack of evidence and research on the integration of livelihood programmes into HIV programmes [38] could be one of the reasons why these programmes have not been more widely implemented. Further implementation research on how these programmes should be evaluated, and subsequent cost-effectiveness studies are needed.

Previous studies [19,20] have also found that being away from home for funerals and travelling (and running out of ART while travelling) were common reasons for missing ART. In our study about 1% of all participants included, or 3% of participants ever missing ART, reported this as a reason for missing ART. Currently cities and urban areas bear a major part of the global HIV burden—in sub-Saharan Africa, nearly half (45%) of people living with HIV reside in urban areas.[39] Travelling and migration in and out of cities can be a challenge for retention in care and adherence but also an important factor linking different networks of HIV transmission.[40] Interventions to provide patients with an adequate supply of ART are critical to minimizing treatment interruptions during travel.

Lack of money for transport was mentioned by 12% of the participants who ever reported missing ART and no transport to the pharmacy by 11%. This has also been cited in other studies as a risk factor for missed medical appointments at the health facility for follow-up and refill because of competing demands between transport costs and other necessities such as food, housing, and school fees.[30,41] Further decentralization of ART services, thereby bringing ART services closer to the patients [42], and involving patients and their families as the model of Community ART Groups (peer support) has the potential to further reduce transport costs and thus minimize out-of-pocket payments.[43] In addition, reducing the frequency of follow-up visits for refills or for routine clinical monitoring may also contribute to reducing the cost burden of transportation.

A large majority of patients (88%) in this study reported experiencing at least one symptom from the HIV Symptom Index, and, not surprisingly, the odds of incomplete adherence increased significantly with each additional symptom, as similarly described elsewhere.[44] Patients who reported feeling sick and uncomfortable because of ART were about 4 times more likely to have incomplete adherence, also confirming findings from other studies.[19,45] We also found that women reported a significantly greater symptom burden (both more symptoms and severity), as described elsewhere.[46]

Fatigue or loss of energy was the most reported symptom (37%), twice as much as reported by Bhatt et. al in South Africa [19], but only attributed to ART by 19% of patients with fatigue. On the other hand, pain, numbness, or tingling in the hands or feet was reported by 35% of patients, and about one third of the patients who reported this symptom attributed it to ART. This is comparable with findings from Thailand where 10% and 28% of probable and possible HIV-associated neuropathy was reported.[47] Peripheral neuropathies are expected to be more prevalent in settings where d4T is part of the first-line regimen.[48] Fortunately, the latest WHO recommendations call for the phasing out of d4T-containing regimens and their replacement by preferably tenofovir-containing regimens.[27] In this study, there was already clear evidence of this change with the proportion of d4T-containing regimens decreasing from 37% at baseline to 18% at the time of the interview, and tenofovir-containing regimens increasing from 11% (at baseline) to 26% (at the time of the interview).

About one third of the patients (30%) reported feeling sad, down, or depressed in the previous four weeks based on the HIV Symptom Index. While we cannot conclude that a patient is suffering from depression based on just one question, this number is surprisingly high and is consistent with the report of moderate to severe depression symptom severity among people living with HIV in sub-Saharan Africa. A systematic review, including 23 studies in sub-Saharan Africa, found prevalence estimates of 18% for major depression and 30% for depression symptoms among HIV-positive patients on ART.[49] In their analysis, patients who reported depression symptoms were 55% less likely to achieve good adherence compared to those not reporting depression symptoms. Similarly, in our univariate analysis, participants who reported feeling sad, down, or depressed were about 50% more likely to report incomplete adherence. This finding supports the screening for depression among patients with HIV.[49] However the region’s health system capacity to detect and treat depression is limited.[50] Dietary protein supplementation has been suggested as a specific strategy to further reduce depression in patients on ART, in settings with food insecurity.[29]

Only one-third of participants reported ever missing ART, which may be low given that other studies have found up to 20% of participants missed taking their ART over just the past week alone.[51] It is well-established that self-report surveys tend to overestimate actual adherence.[52] However, this overestimate may also introduce a bias by not capturing the reasons for missed ART among both people willing to disclose, as well as among people unwilling to disclose incomplete adherence behaviors.

This study was conducted in 2011 before the WHO guidelines to replace d4T-containing regiments with tenofovir-containing regiments were introduced. [27] As such the factors related to missing ART may differ among patients on tenofovir-containing regimens.

We found some variability in the proportion of symptoms attributed to ART by patients: symptoms of peripheral neuropathy, troubles with remembering, nausea and vomiting, skin problems and problems with having sex were mostly attributed to ART. This could potentially lead to some bias with less adherent individuals more likely to report symptoms and attribute them to their ART. The cross-sectional design of the study limits the causal inference of whether it is incomplete adherence that leads to symptoms or whether it is the symptoms that are leading to incomplete adherence. Another limitation of the study is the difficulty in attributing symptoms to ART; some of these symptoms may also arise from the HIV infection itself, or arise from co- morbidities frequently associated with HIV infection (diabetes, hepatitis C infection).[53] We were also unable to assess whether ART clients who declined to participate differed from those participating in the study.

## Conclusions

Symptoms were a common reason for missing ART, together with simply forgetting and food insecurity. Women and participants taking ART regimens containing d4T at initiation experienced greater symptom burden. A combination of ART regimens with fewer side effects, use of mobile phone text messaging, and integration of food supplementation and livelihood programmes into HIV programmes, have the potential to decrease missed doses of ART and hence to improve adherence and the outcomes of ART programmes.

## References

[1] UNAIDS. The gap report 2014. 2014. Available: http://www.unaids.org/sites/default/files/en/media/unaids/contentassets/documents/unaidspublication/2014/UNAIDS_Gap_report_en.pdf. Accessed 22 July 2015.

[2] UNAIDS. Ambitious treatment targets: writing the final chapter on the AIDS epidemic. 2014. Available: http://www.unaids.org/sites/default/files/media_asset/JC2670_UNAIDS_Treatment_Targets_en.pdf. Accessed 22 July 2015.

[3] Cohen MS, Chen YQ, McCauley M, Gamble T, Hosseinipour MC, Kumarasamy N, et al Prevention of HIV-1 infection with early antiretroviral therapy. N Engl J Med 2011 8 11;365(6):493–505. 10.1056/NEJMoa1105243 21767103 PMC3200068

[4] Loutfy MR, Wu W, Letchumanan M, Bondy L, Antoniou T, Margolese S, et al Systematic review of HIV transmission between heterosexual serodiscordant couples where the HIV-positive partner is fully suppressed on antiretroviral therapy. PLOS ONE 2013;8(2):e55747 Available: http://journals.plos.org/plosone/article?id=10.1371/journal.pone.0055747 Accessed: 2015 July 22. 10.1371/journal.pone.0055747 23418455 PMC3572113

[5] McMahon JH, Elliott JH, Bertagnolio S, Kubiak R, Jordan MR. Viral suppression after 12 months of antiretroviral therapy in low- and middle-income countries: a systematic review. Bull World Health Organ 2013 5 1;91(5):377–385E. Available: http://www.who.int/bulletin/volumes/91/5/12-112946.pdf.Accessed 22 July 2015. 10.2471/BLT.12.112946 23678201 PMC3646348

[6] Hogg RS, Heath K, Bangsberg D, Yip B, Press N, O'Shaughnessy MV, et al Intermittent use of triple-combination therapy is predictive of mortality at baseline and after 1 year of follow-up. AIDS 2002 5 3;16(7):1051–8. 11953472 10.1097/00002030-200205030-00012

[7] Chesney MA. Factors affecting adherence to antiretroviral therapy. Clin Infect Dis 2000 6;30 Suppl 2:S171–S176. 10860902 10.1086/313849

[8] Mills EJ, Nachega JB, Bangsberg DR, Singh S, Rachlis B, Wu P, et al Adherence to HAART: a systematic review of developed and developing nation patient-reported barriers and facilitators. PLOS Med 2006 11;3(11):e438 Available: http://www.plosmedicine.org/article/Related/info:doi/10.1371/journal.pmed.0030438. Accessed 22 July 2015. 17121449 10.1371/journal.pmed.0030438 PMC1637123

[9] Denison JA, Koole O, Tsui S, Menten J, Torpey K, van PE, et al Incomplete adherence among treatment-experienced adults on antiretroviral therapy in Tanzania, Uganda and Zambia. AIDS 2015 1 28;29(3):361–71. 10.1097/QAD.0000000000000543 25686684 PMC4728173

[10] Koole O, Tsui S, Wabwire-Mangen F, Kwesigabo G, Menten J, Mulenga M, et al Retention and risk factors for attrition among adults in antiretroviral treatment programmes in Tanzania, Uganda and Zambia. Trop Med Int Health 2014 12;19(12):1397–410. 10.1111/tmi.12386 25227621 PMC4724698

[11] Kalichman SC, Simbayi LC, Cloete A, Mthembu PP, Mkhonta RN, Ginindza T (2009) Measuring AIDS stigmas in people living with HIV/AIDS: the Internalized AIDS-Related Stigma Scale. AIDS Care 21: 87–93. 10.1080/09540120802032627 19085224

[12] Kaaya SF, Fawzi MC, Mbwambo JK, Lee B, Msamanga GI, Fawzi W (2002) Validity of the Hopkins Symptom Checklist-25 amongst HIV-positive pregnant women in Tanzania. Acta Psychiatr Scand 106: 9–19. 12100343 10.1034/j.1600-0447.2002.01205.x PMC6300056

[13] Broadhead WE, Gehlbach SH, de Gruy FV, Kaplan BH (1988) The Duke-UNC Functional Social Support Questionnaire. Measurement of social support in family medicine patients. Med Care 26: 709–723. 3393031 10.1097/00005650-198807000-00006

[14] Samet JH, Phillips SJ, Horton NJ, Traphagen ET, Freedberg KA (2004) Detecting alcohol problems in HIV-infected patients: use of the CAGE questionnaire. AIDS Res Hum Retroviruses 20: 151–155. 15018702 10.1089/088922204773004860

[15] Chesney MA, Ickovics JR, Chambers DB, Gifford AL, Neidig J, Zwickl B, et al Self-reported adherence to antiretroviral medications among participants in HIV clinical trials: the AACTG adherence instruments. Patient Care Committee & Adherence Working Group of the Outcomes Committee of the Adult AIDS Clinical Trials Group (AACTG). AIDS Care 2000 6;12(3):255–66. 10928201 10.1080/09540120050042891

[16] Justice AC, Holmes W, Gifford AL, Rabeneck L, Zackin R, Sinclair G, et al Development and validation of a self-completed HIV symptom index. J Clin Epidemiol 2001 12;54 Suppl 1:S77–S90. 11750213 10.1016/s0895-4356(01)00449-8

[17] Oyugi JH, Byakika-Tusiime J, Ragland K, Laeyendecker O, Mugerwa R, Kityo C, et al Treatment interruptions predict resistance in HIV-positive individuals purchasing fixed-dose combination antiretroviral therapy in Kampala, Uganda. AIDS 2007 5 11;21(8):965–71. 17457090 10.1097/QAD.0b013e32802e6bfa

[18] Genberg BL, Wilson IB, Bangsberg DR, Arnsten J, Goggin K, Remien RH, et al Patterns of antiretroviral therapy adherence and impact on HIV RNA among patients in North America. AIDS 2012 7 17;26(11):1415–23. 10.1097/QAD.0b013e328354bed6 22767342 PMC3655551

[19] Bhat VG, Ramburuth M, Singh M, Titi O, Antony AP, Chiya L, et al Factors associated with poor adherence to anti-retroviral therapy in patients attending a rural health centre in South Africa. Eur J Clin Microbiol Infect Dis 2010 8;29(8):947–53. 10.1007/s10096-010-0949-4 20467769

[20] Unge C, Sodergard B, Marrone G, Thorson A, Lukhwaro A, Carter J, et al Long-term adherence to antiretroviral treatment and program drop-out in a high-risk urban setting in sub-Saharan Africa: a prospective cohort study. PLOS ONE 2010;5(10):e13613 Available: http://journals.plos.org/plosone/article?id=10.1371/journal.pone.0013613 Accessed: 2015 July 22. 10.1371/journal.pone.0013613 21049045 PMC2963610

[21] Barfod TS, Sorensen HT, Nielsen H, Rodkjaer L, Obel N. 'Simply forgot' is the most frequently stated reason for missed doses of HAART irrespective of degree of adherence. HIV Med 2006 7;7(5):285–90. 16945072 10.1111/j.1468-1293.2006.00387.x

[22] Lester RT, Ritvo P, Mills EJ, Kariri A, Karanja S, Chung MH, et al Effects of a mobile phone short message service on antiretroviral treatment adherence in Kenya (WelTel Kenya1): a randomised trial. Lancet 2010 11 27;376(9755):1838–45. 10.1016/S0140-6736(10)61997-6 21071074

[23] Roux P, Kouanfack C, Cohen J, Marcellin F, Boyer S, Delaporte E, et al Adherence to antiretroviral treatment in HIV-positive patients in the Cameroon context: promoting the use of medication reminder methods. J Acquir Immune Defic Syndr 2011 7 1;57 Suppl 1:S40–S43. 10.1097/QAI.0b013e318222b5c2 21857285

[24] Finitsis DJ, Pellowski JA, Johnson BT. Text message intervention designs to promote adherence to antiretroviral therapy (ART): a meta-analysis of randomized controlled trials. PLOS ONE 2014;9(2):e88166 Available: http://journals.plos.org/plosone/article?id=10.1371/journal.pone.0088166. Accessed 22 July 2015. 10.1371/journal.pone.0088166 24505411 PMC3914915

[25] Mills EJ, Lester R, Thorlund K, Lorenzi M, Muldoon K. Interventions to promote adherence to antiretroviral therapy in Africa: a network meta-analysis. Lancet HIV 2014 12;1(3):e104–e111. 10.1016/S2352-3018(14)00003-4 26424119 PMC5096455

[26] Thirumurthy H, Lester RT. M-health for health behaviour change in resource-limited settings: applications to HIV care and beyond. Bull World Health Organ 2012 5 1;90(5):390–2. Available: http://www.who.int/bulletin/volumes/90/5/11-099317/en/. Accessed 22 July 2015. 10.2471/BLT.11.099317 22589574 PMC3341690

[27] WHO. Consolidated guidelines on the use of antiretroviral drugs for treating and preventing HIV infection. Recommendations for a public health approach 2013.2013. Available: http://apps.who.int/iris/bitstream/10665/85321/1/9789241505727_eng.pdf?ua=1. Accessed 22 July 2015. 24716260

[28] Rodrigues R, Bogg L, Shet A, Kumar DS, De CA. Mobile phones to support adherence to antiretroviral therapy: what would it cost the Indian National AIDS Control Programme? J Int AIDS Soc 2014;17:19036 Available: http://www.jiasociety.org/index.php/jias/article/view/19036/3934. Accessed 22 July 2015. 10.7448/IAS.17.1.19036 25186918 PMC4154142

[29] Martinez P, Tsai AC, Muzoora C, Kembabazi A, Weiser SD, Huang Y, et al Reversal of the Kynurenine pathway of tryptophan catabolism may improve depression in ART-treated HIV-infected Ugandans. J Acquir Immune Defic Syndr 2014 4 1;65(4):456–62. 10.1097/QAI.0000000000000062 24220289 PMC3943704

[30] Hardon AP, Akurut D, Comoro C, Ekezie C, Irunde HF, Gerrits T, et al Hunger, waiting time and transport costs: time to confront challenges to ART adherence in Africa. AIDS Care 2007 5;19(5):658–65. 17505927 10.1080/09540120701244943

[31] Musumari PM, Feldman MD, Techasrivichien T, Wouters E, Ono-Kihara M, Kihara M. "If I have nothing to eat, I get angry and push the pills bottle away from me": A qualitative study of patient determinants of adherence to antiretroviral therapy in the Democratic Republic of Congo. AIDS Care 2013;25(10):1271–7. 10.1080/09540121.2013.764391 23383757

[32] Musumari PM, Wouters E, Kayembe PK, Kiumbu NM, Mbikayi SM, Suguimoto SP, et al Food insecurity is associated with increased risk of non-adherence to antiretroviral therapy among HIV-infected adults in the Democratic Republic of Congo: a cross-sectional study. PLOS ONE 2014;9(1):e85327 Available: http://journals.plos.org/plosone/article?id=10.1371/journal.pone.0085327. Accessed 22 July 2015. 10.1371/journal.pone.0085327 24454841 PMC3893174

[33] Singer AW, Weiser SD, McCoy SI. Does Food Insecurity Undermine Adherence to Antiretroviral Therapy? A Systematic Review. AIDS Behav 2014 8 6. 10.1007/s10461-014-0873-1 25096896

[34] Weiser SD, Fernandes KA, Brandson EK, Lima VD, Anema A, Bangsberg DR, et al The association between food insecurity and mortality among HIV-infected individuals on HAART. J Acquir Immune Defic Syndr 2009 11 1;52(3):342–9. 10.1097/QAI.0b013e3181b627c2 19675463 PMC3740738

[35] Weiser SD, Palar K, Frongillo EA, Tsai AC, Kumbakumba E, Depee S, et al Longitudinal assessment of associations between food insecurity, antiretroviral adherence and HIV treatment outcomes in rural Uganda. AIDS 2014 1 2;28(1):115–20. 10.1097/01.aids.0000433238.93986.35 23939234 PMC4629837

[36] Cantrell RA, Sinkala M, Megazinni K, Lawson-Marriott S, Washington S, Chi BH, et al A pilot study of food supplementation to improve adherence to antiretroviral therapy among food-insecure adults in Lusaka, Zambia. J Acquir Immune Defic Syndr 2008 10 1;49(2):190–5. 10.1097/QAI.0b013e31818455d2 18769349 PMC3847664

[37] Mamlin J, Kimaiyo S, Lewis S, Tadayo H, Jerop FK, Gichunge C, et al Integrating nutrition support for food-insecure patients and their dependents into an HIV care and treatment program in Western Kenya. Am J Public Health 2009 2;99(2):215–21. 10.2105/AJPH.2008.137174 19059851 PMC2622780

[38] Yager JE, Kadiyala S, Weiser SD. HIV/AIDS, food supplementation and livelihood programs in Uganda: a way forward? PLOS ONE 2011;6(10):e26117 Available: http://journals.plos.org/plosone/article?id=10.1371/journal.pone.0026117. Accessed 22 July 2015. 10.1371/journal.pone.0026117 22022530 PMC3192151

[39] UNAIDS. The Cities Report. 2014. Available: http://www.unaids.org/sites/default/files/media_asset/JC2687_TheCitiesReport_en.pdf. Accessed 22 July 2015.

[40] Voeten HA, Vissers DC, Gregson S, Zaba B, White RG, de Vlas SJ, et al Strong association between in-migration and HIV prevalence in urban sub-Saharan Africa. Sex Transm Dis 2010 4;37(4):240–3. 10.1097/OLQ.0b013e3181c3f2d0 19959971 PMC3514976

[41] Tuller DM, Bangsberg DR, Senkungu J, Ware NC, Emenyonu N, Weiser SD. Transportation costs impede sustained adherence and access to HAART in a clinic population in southwestern Uganda: a qualitative study. AIDS Behav 2010 8;14(4):778–84. 10.1007/s10461-009-9533-2 19283464 PMC2888948

[42] Koole O, Houben RM, Mzembe T, Van Boeckel TP, Kayange M, Jahn A, et al Improved retention of patients starting antiretroviral treatment in Karonga District, northern Malawi, 2005–2012. J Acquir Immune Defic Syndr 2014 9 1;67(1):e27–e33. 10.1097/QAI.0000000000000252 24977375 PMC4240943

[43] Rasschaert F, Telfer B, Lessitala F, Decroo T, Remartinez D, Biot M, et al A qualitative assessment of a community antiretroviral therapy group model in tete, mozambique. PLOS ONE 2014;9(3):e91544 Available: http://journals.plos.org/plosone/article?id=10.1371/journal.pone.0091544. Accessed 22 July 2015. 10.1371/journal.pone.0091544 24651523 PMC3961261

[44] Duran S, Spire B, Raffi F, Walter V, Bouhour D, Journot V, et al Self-reported symptoms after initiation of a protease inhibitor in HIV-infected patients and their impact on adherence to HAART. HIV Clin Trials 2001 1;2(1):38–45. 11590513 10.1310/R8M7-EQ0M-CNPW-39FC

[45] Al-Dakkak I, Patel S, McCann E, Gadkari A, Prajapati G, Maiese EM. The impact of specific HIV treatment-related adverse events on adherence to antiretroviral therapy: a systematic review and meta-analysis. AIDS Care 2013;25(4):400–14. 10.1080/09540121.2012.712667 22908886 PMC3613968

[46] Bonfanti P, Valsecchi L, Parazzini F, Carradori S, Pusterla L, Fortuna P, et al Incidence of adverse reactions in HIV patients treated with protease inhibitors: a cohort study. Coordinamento Italiano Studio Allergia e Infezione da HIV (CISAI) Group. J Acquir Immune Defic Syndr 2000 3 1;23(3):236–45. 10839659 10.1097/00126334-200003010-00004

[47] Sithinamsuwan P, Punthanamongkol S, Valcour V, Onsanit S, Nidhinandana S, Thitivichianlert S, et al Frequency and characteristics of HIV-associated sensory neuropathy among HIV patients in Bangkok, Thailand. J Acquir Immune Defic Syndr 2008 12 1;49(4):456–8. 10.1097/QAI.0b013e318186eb03 19011422 PMC3635535

[48] Sacktor N, Nakasujja N, Skolasky RL, Robertson K, Musisi S, Ronald A, et al Benefits and risks of stavudine therapy for HIV-associated neurologic complications in Uganda. Neurology 2009 1 13;72(2):165–70. 10.1212/01.wnl.0000339042.96109.86 19139369 PMC2677497

[49] Nakimuli-Mpungu E, Bass JK, Alexandre P, Mills EJ, Musisi S, Ram M, et al Depression, alcohol use and adherence to antiretroviral therapy in sub-Saharan Africa: a systematic review. AIDS Behav 2012 11;16(8):2101–18. 10.1007/s10461-011-0087-8 22116638

[50] WHO. mhGAP: Mental Health Gap Action Programme. 2008. Available: http://whqlibdoc.who.int/publications/2008/9789241596206_eng.pdf?ua=1. Accessed 22 July 2015.

[51] Sarna A, Pujari S, Sengar AK, Garg R, Gupta I, Dam J (2008) Adherence to antiretroviral therapy & its determinants amongst HIV patients in India. Indian J Med Res 127: 28–36. 18316850

[52] Haubrich RH, Little SJ, Currier JS, Forthal DN, Kemper CA, Beall GN, Johnson D, Dube MP, Hwang JY, McCutchan JA (1999) The value of patient-reported adherence to antiretroviral therapy in predicting virologic and immunologic response. California Collaborative Treatment Group. AIDS 13: 1099–1107. 10397541 10.1097/00002030-199906180-00014

[53] Nachega JB, Trotta MP, Nelson M, Ammassari A. Impact of metabolic complications on antiretroviral treatment adherence: clinical and public health implications. Curr HIV /AIDS Rep 2009 8;6(3):121–9. 19589297 10.1007/s11904-009-0017-9

